# Putative stem cells and epithelial-mesenchymal transition revealed in sections of ovarian tumor in patients with serous ovarian carcinoma using immunohistochemistry for vimentin and pluripotency-related markers

**DOI:** 10.1186/s13048-017-0306-7

**Published:** 2017-02-23

**Authors:** Natasa Kenda Suster, Spela Smrkolj, Irma Virant-Klun

**Affiliations:** Department of Obstetrics and Gynaecology, University Medical Centre Ljubljana, Slajmerjeva 3, 1000 Ljubljana, Slovenia

**Keywords:** Ovarian cancer, Epithelial-mesenchymal transition, Cancer stem cells, Markers of pluripotency, Immunohistochemistry

## Abstract

**Background:**

The mechanism of aggressive character of ovarian cancer and unsuccessful treatment of women with this deadly disease has been recently explained by the theory of cancer stem cells (CSCs). It has been reported that ovarian carcinogenesis and progression of disease is associated with epithelial-mesenchymal transition (EMT). EMT, a physiological cell process during embryonic development and later in life during regeneration, could, when induced in pathological condition, generate CSCs-like cells. Until now EMT in the ovarian tissue has been mainly studied in cell cultures in vitro. The aim of this study was to focus on in situ morphological changes in the ovarian surface epithelium of tumor tissue in women with epithelial ovarian cancer after we applied the antibodies for markers of EMT vimentin and pluripotency-related markers NANOG, SOX2 and SSEA-4.

**Methods:**

We analyzed ovarian tissue sections of 20 women with high grade serous ovarian carcinoma. After eosin and hematoxylin staining, used in standard practice, immunohistochemistry was performed for vimentin and markers of pluripotency: NANOG, SSEA-4 and SOX2. We focused on the ovarian surface epithelium in order to observe morphological changes in tumor tissue.

**Results:**

Among epithelial cells of the ovarian surface epithelium in women with serous ovarian carcinoma we observed a population of small NANOG-positive cells with diameters of up to 5 μm and nuclei, which filled almost the entire cell volumes. These small NANOG-positive cells were in some cases concentrated in the regions with morphologically changed epithelial cells. In these regions, a population of bigger round cells with diameters of 10–15 μm with large nuclei, and positively stained for vimentin, NANOG and other markers of pluripotnecy, were released from the surface epithelium. These cells are proposed as CSCs, and possibly originate from small stem cells among epithelial cells. They formed typical cell clusters, invaded the tissue by changing their round shape into a mesenchymal-like phenotype, and contributed to the manifestation of ovarian cancer.

**Conclusions:**

Our findings show morphological changes in the ovarian surface epithelium in tumor slides of high grade serous ovarian carcinoma and provide a new population of putative CSCs.

**Electronic supplementary material:**

The online version of this article (doi:10.1186/s13048-017-0306-7) contains supplementary material, which is available to authorized users.

## Background

Ovarian cancer is the second most common gynecological cancer and the leading cause of death among all gynecological tumors; the incidence in our country is 16 per 100,000 females [1]. Approximately 90% of ovarian cancers belong to the group of epithelial ovarian cancers. The majority, 75% of epithelial ovarian cancers, are of the serous histologic type. Due to an insufficient screening program for ovarian cancer and a lack of early specific symptoms, it is difficult to make an early diagnosis [2]. Consequently, the majority of patients with ovarian cancer (77.8%) are at the advanced stage, with metastatic sites disseminated widely within the peritoneal cavity [1], whereby the time for successful (complete cytoreductive) surgery has already been missed. Although the majority of these patients respond well to standard chemotherapy, achieving remission of the disease, unfortunately, in over 70% of patients the tumor relapses in time, resulting in a less than 30% 5-year survival rate [1].

Despite intensive research on ovarian cancer, its manifestation is still poorly understood. The mechanism of the aggressive character of ovarian cancer has recently been explained by the theory of cancer stem cells (CSC). CSCs maintain their undifferentiated state and the ability of self-renewal on one hand and have the ability of differentiation, invasion and metastases on the other hand. Until now, the phenotype and molecular status of population of ovarian cancer tumor-initiating stem cells have not been defined. Even more, the CSC phenotype may not be uniform among different cancer types and subtypes or even in tumors of the same histological types or subtypes [3]. Heterogeneity within tumor cells may lead to a different course of disease, a different response to treatment, and drug resistance [4].

Nanog homeobox (NANOG) is a transcription factor, which, along with transcription factors octamer-binding protein 4 (OCT4) and SRY-box 2 (SOX2), plays a key role in the maintenance of pluripotency and self-renewal in undifferentiated embryonic stem cells (ESCs) [5–9]. The surface antigen SSEA-4 (stage-specific embryonic antigen 4) is another pluripotent stem cell marker. Its expression was found in the epithelial ovarian cancer [10]. NANOG has also been detected in different types of tumors, including the epithelial ovarian cancer [9, 11–13]. Even more, the overexpression of NANOG in the epithelial ovarian cancer was associated with a high grade tumor, advanced clinical stage of disease [13–15], resistance to chemotherapy [14], and shorter patient survival rate [14, 15]. The most recent discoveries have also showed that NANOG regulates the epithelial-mesenchymal transition (EMT) and chemoresistance through activation of the signal transducer and activator of transcription 3 (STAT3) pathway [16].

The association of EMT with ovarian carcinogenesis and progression was also reported by other studies [17–19]. EMT is a physiological cell reprogramming event utilized in tissue remodeling during the embryonic development and is activated in normal adult tissues during regeneration. EMT is defined as the loss of epithelial traits by the former epithelial cells with acquisition of mesenchymal characteristics, such as invasive motility and the presence of vimentin and myosin [20]. During EMT unique characteristics of certain mesenchymal cells are acquired, including epithelial cell polarity, intracellular adhesion and loss of specific cell surface markers. Due to cytoskeletal remodeling, these cells subsequently obtain a mesenchymal-like phenotype. The major molecular characteristics of EMT are the downregulation of epithelial cell markers, E-cadherin and ß-catenin, and upregulation of the markers of mesenchymal phenotype, vimentin, fibronectin and N-cadherin. Recent findings suggest that EMT, induced in pathological condition, could generate ovarian CSCs-like cells [21]. EMT in epithelial tumor cells increases their invasive capacity and enhances their degree of malignancy [22, 23]. EMT is also considered a key step in CSC metastases [24]. During EMT, carcinoma cells lose their epithelial characteristics and acquire mesenchymal properties that promote extracellular matrix invasion and distant metastases. Several molecular mechanisms are involved in this process, like down-regulation of E-cadherin, cytokeratins, zona occludens 1 (ZO-1), claudins, occludin, laminin-1, entactin, mucin 1 (MUC-1), the microRNA 200 family, and acquisition of the transcription factors Snail 1, Snail 2, Twist, Zeb1 and Zeb2/SIP1 as well as N-cadherin, fibronectin, vimentin, and others [25, 26]. The major cytoskeletal component of mesenchymal cells is vimentin, which is usually used as a marker of mesenchymally-derived cells or cells undergoing an EMT during metastatic progression, and is related to primary (intrinsic) resistance or poor response to chemotherapy and metastasis promotion [27, 28]. Tumor cells undergoing EMT acquire the capacity to disarm the body's antitumor defense, resist apoptosis and anticancer drugs [28]. Transformed tumor cells disseminate throughout the organism, and act as a reservoir that refills and expands the tumor cell population [29]. As EMT represents a key event in cancer manifestation and progression, it is becoming a promising target for anticancer therapy [29, 30].

Until now, EMT has been studied mainly in normal ovarian cell cultures and ovarian tumor cell cultures in vitro, but rarely in situ on tumor tissue. The aim of this study was to apply the antibodies for marker of EMT vimentin and markers of pluripotency NANOG, SOX2 and SSEA-4 to ovarian tumor sections of women with epithelial cancer, and focus on morphological changes in the ovarian surface epithelium. We found a population of round cells forming and releasing from the ovarian surface epithelium that were vimentin and NANOG-positive. A similar population of cells was also identified when SOX2 and SSEA-4 markers of pluripotency were applied. We suggest these cells to be involved in EMT and manifestation of ovarian cancer. These cells need to be further researched.

## Methods

We analyzed ovarian tumor samples of 20 women with high-grade serous ovarian carcinoma. Patients were surgically treated in the Department of Gynecology and Obstetrics, University Medical Centre Ljubljana, Slovenia, and tumor samples were collected at the Histopathology Unit to carry out the histopathological diagnosis and were then included in our study. Tumor samples were collected before the patients were treated with chemotherapy. All tumor samples were reviewed by a single pathologist. Ovarian tissue sections were stained by hematoxylin and eosin (HE), common in the daily medical practice, which resulted in blue-stained cell nuclei. Additionally, immunohistochemistry (IHC) was used for analyzing the marker of EMT vimentin, and pluripotency-related marker NANOG to identify potential stem cells expressing this marker and to analyze their localization in the ovarian surface epithelium. In three women with high grade serous ovarian carcinoma, the ovarian sections were further stained for pluripotency-related markers SSEA-4 and SOX2. In our study, we focused particularly on cells in the ovarian surface epithelium that were expressing vimentin and a degree of pluripotency.

### Immunohistochemistry for vimentin

IHC analysis for vimentin was performed on tissue microarray. 3-5 μm sections of tumor tissue were cut from the same multitumor block as in the case of NANOG IHC analysis. The 3-5 μm-thick paraffin sections were placed on silane-coated slides (Menzel-Glaser Superfrost) and dried in a dryer at a temperature of 60 °C for one hour. IHC staining was performed by an automatic slide stainer (Ventana BenchMark GX). After deparaffinization antigen retrieval (HIER) took place at pH 7-8 for 48 min with reagent CC1 Ventana. Slides were then incubated with a monoclonal mouse anti-vimentin antibody, clone V9, DakoCytomation, at dilution 1: 300 for 30 min at room temperature. The antigen detection was performed with the Ventana OptiView Kit. The removal of the primary antibody served as a negative control. The stained ovarian sections were monitored under light, inverted, and fluorescence microscope. The cells were supposed to be positive if they featured a brown stain.

### Immunohistochemistry for NANOG

IHC analysis was performed on a tissue microarray (TMA) of tumor samples of serous ovarian carcinoma. After evaluation of HE stained sections of the ovarian tumor, the biopsy of representative areas of the tumor tissue was performed with a 2 mm core needle. The biopsy specimens were then transferred to multitumor paraffin blocks. 3-5 μm sections were consecutively cut from the multitumor block. The 3-5 μm thick paraffin sections were then placed on silane-coated slides (Menzel-Glaser Superfrost) and were dried for one hour in a dryer at 60 °C. IHC staining was performed by an automatic slide stainer (Ventana BenchMark GX). After deparaffinization, the antigen retrieval was performed. Antigen retrieval (HIER) was performed with a CC1 Ventana reagent at pH 7-8 for 48 min. The primary antibody was then applied. Rabbit anti-human NANOG monoclonal antibody (ab109250, Abcam Cambridge, MA, USA) was used as the primary antibody at a dilution rate of 1:25. Incubation with NANOG antibody lasted for 30 min at 37 °C. The antigen detection was performed with the Ventana OptiView Kit. The removal of the primary antibody served as a negative control. The stained slides were observed under microscope. The positive reaction of the NANOG protein was visible as yellow-brown staining of the nuclei of cells of the ovarian serous carcinoma.

### Immunohistochemistry for SSEA-4 and SOX2

The paraffin embedded ovarian tissue was cut into 4 μm-thick sections which were then placed on silane-coated slides and dried overnight at 37 °C. The next morning the sections were deparaffinised and rehydrated through an ethanol series. Antigen retrieval was performed in Target Retrieval Solution. The sections were boiled in Target Retrieval Solution in a microwave oven (Dako) at low power for 10 min. After cooling down, the sections were rinsed with phosphate buffered saline (PBS). When intracellular antigen was observed, the sections were permeabilized with 0.3% Triton X-100 for 10 min and then rinsed with PBS. Unspecific binding sites were blocked with 10% fetal bovine serum. The tissue sections were then incubated for 2 h in the mouse anti-SSEA-4 fluorescein isothiocynate (FITC)-conjugated antibodies at a dilution rate of 1:200 or mouse anti-SOX2 phycoerythrin (PE)-conjugated antibodies at a dilution rate of 1:100 (both BD Biosciences). The primary antibody was removed for negative control. After rinsing the tissue sections with PBS, the slides were mounted with the Vectashield mounting medium with 4',6-diamidino-2-phenylindole (DAPI) (VectorLaboratories). The stained slides were observed under a fluorescent microscope. After DAPI staining, the cell nuclei were stained blue. Moreover, the SSEA-4-positive cells expressed surface green fluorescence and SOX2-positive cells expressed nuclear red fluorescence. We focused on SSEA-4 surface green fluorescence staining and SOX2-nuclei red fluorescence staining, matching DAPI blue nuclei staining.

The stained slides were monitored under a microscope. Light microscope (LM) was used at magnifications up to 1000-times, and in order to better elucidate the potential stem cells an inverted microscope was used at magnifications up to 200-times.

## Results

### Vimentin-positive round cells releasing from ovarian surface epithelium

**Fig. 1:**
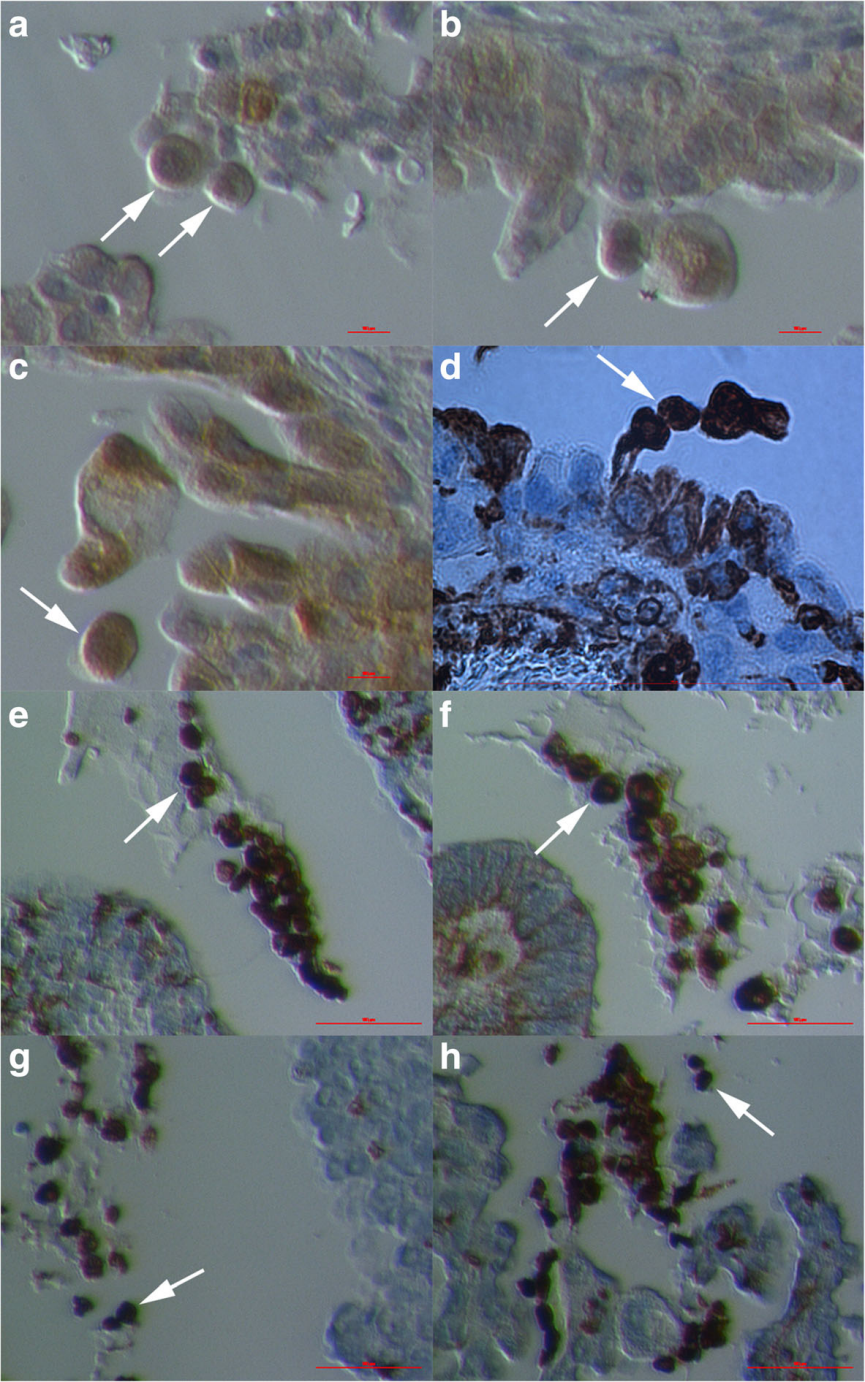
Round vimentin-positive cells (*arrows*) with diameters of 10–15 μm, which were released from the ovarian surface epithelium (OSE). Round cells, which were slightly positive for vimentin, expressed big nuclei and were being released from the epithelium (**a**, **b**). After release from OSE, these round cells became strongly positive for vimentin (**c**, **d**). Some of them still had some cytoplasmic residual after release and were connected (**d**). Typical clusters formed from round cells, which released from OSE and were positively-stained for vimentin and embedded by a kind of matrix (**e**–**h**). (Inverted microscope: **a**–**c**, **e**–**h**, magnifications 100x and 200x; light microscope: **d**, magnification: 400x) *Legend*: *brown*-vimentin positivity and *blue*-nuclei after HE staining. *Red Bar*: 10 μm for **a**-**c** and 100 μm for **d**–**h**

After immunohistochemistry for vimentin we found a population of round cells being released from the ovarian surface epithelium (OSE). The majority of these cells had diameters of 10–15 μm, were slightly positive for vimentin and were being released from OSE (Fig. 1a, b and Additional file 1: Figure S1). Their nuclei were clearly visible due to HE staining and filled almost the entire volumes of these cells, which is a characteristic of stem cells. Sometimes also the nearby epithelial cells expressed a weak cytoplasmic expression of vimentin (Fig. 1c, d). When round cells were separated from the OSE they became strongly positive for vimentin (Fig. 1c, d) and formed typical cell clusters (Fig. 1e-h), which were embedded by a kind of transparent matrix (Fig. 1e-g). Some of these round cells still had an elongated cytoplasmic residue, which remains from their releasing from the OSE (Fig. 1d). In these typical cell clusters, there were also smaller round cells with diameters of around 5 μm (Fig. 1e-h), which were, similarly to bigger round cells, strongly positive for vimentin; the nuclei filled almost the entire volumes of these small cells. Both bigger and small round cells were completely brown-stained for vimentin (Fig. 1). In some cases the nearby epithelial cells also expressed a weak cytoplasmic expression of vimentin (Fig. 1c, d) but they mostly didn’t.

### Proliferation of vimentin-positive cells

**Fig. 2:**
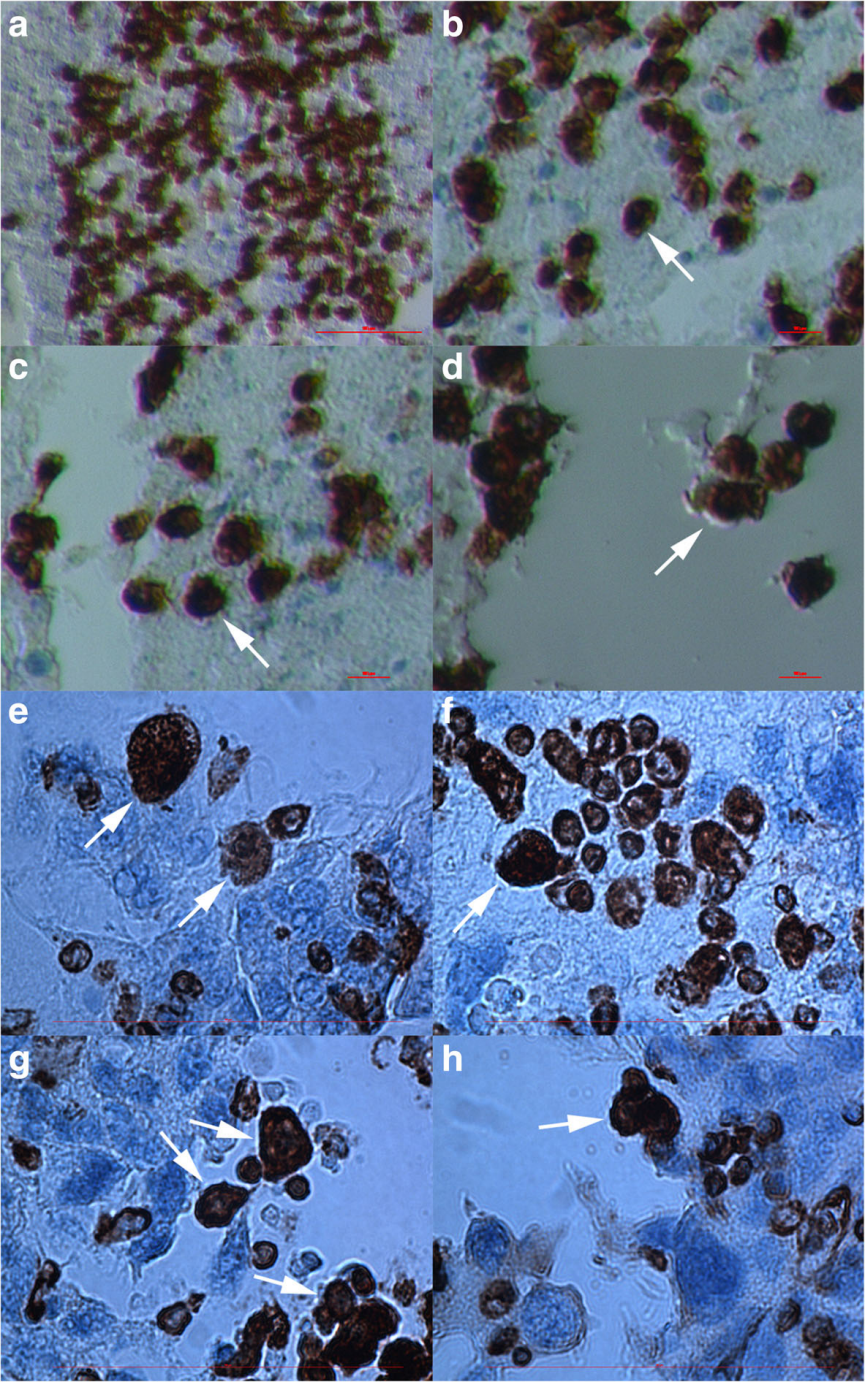
Proliferation of vimentin-positive round cells, which were released from the ovarian surface epithelium. The majority of cells expressed diameters of 10–15 μm (**a**–**d**). However, there were some smaller cells among them that expressed diameters of around 5 μm (**e**–**h**); these smaller cells expressed cytoplasmic positivity for vimentin. Among these smaller cells were bigger vimentin-positive cells (arrows), which formed clusters and possibly originate from smaller cells (**e**–**h**). (Inverted microscope: **a**–**d**, magnifications 100x and 200x; light microscope: **e**–**h**, magnification 400x). *Legend*: *brown*-vimentin positivity and blue-nuclei after HE staining. *Red Bar*: 10 μm for **b**–**d** and 100 μm for **a**, **e**–**h**

When released from the OSE, the round vimentin-positive cells were intensely proliferating (Fig. 2). In some places these cells were proliferating also inside the tissue. The population of proliferating cells was composed of vimentin-positive cells of different diameters (Fig. 2a-d) and in the majority of them the nuclei filled almost the entire cell volumes. In Fig. 2e-h we can see that the proliferating cells included small round cells with diameters around 5 μm, which expressed a cytoplasmic positivity for vimentin; the nuclei filled almost the entire cell volume of these cells and a thin ring of cytoplasm was positively stained for vimentin. These small cells included growing cells and also bigger round cells with diameters of 10–15 μm, which were strongly positively-stained for vimentin and were quite similar to cells released from the OSE (Fig. 2e-h). It is not excluded that small round cells grew into bigger vimentin-positive cells, which also formed clusters (Fig. 2g, h).

### Vimentin-positive cells invaded the ovarian tissue

**Fig. 3:**
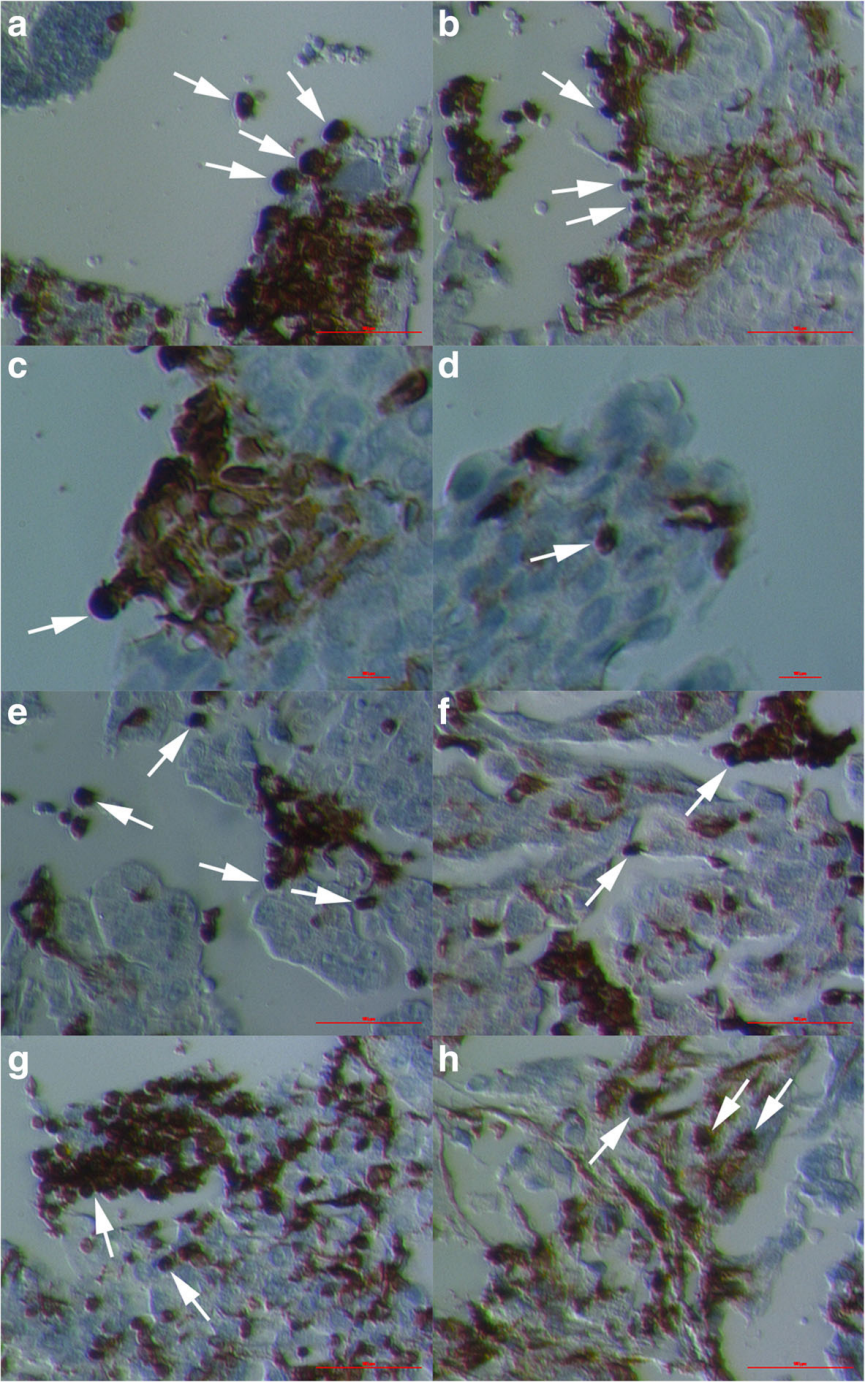
Invasion of round vimentin-positive cells with diameters of 10–15 μm deeper into the ovarian tissue. These cells (*arrows*) were at some places accumulated at the surface of the tissue and progressed deeper into the tissue by their change into the mesenchymal-like phenotype (**a**–**c**) by producing protrusions (**d**–**g**) and elongation (**h**); after changing their shape, they were still positive for vimentin. (Inverted microscope, magnifications 100x and 200x). *Legend*: *brown*-vimentin positivity and *blue*-nuclei after HE staining. *Red Bar*: 10 μm for **c**, **d**, and 100 μm for **a**, **b**, **e**–**h**

In several places of ovarian sections it was possible to see very similar clusters of round vimentin-positive cells with diameters of 10–15 μm much like following the release from OSE, which were invading the ovarian tissue by changing their round shape into mesenchymal-like phenotype with protrusions and elongation (Fig. 3). Moreover, after establishing the mesenchymal-like phenotype, they remained positive for vimentin. The round vimentin-positive cells were accumulated and proliferating at the ovarian tissue surface on several occasions and were invading inside the tissue by making their protrusions and elongation, which can be seen in Fig. 3a-f. These cells remained vimentin positive even after elongation (Fig. 3h).

### Vimentin-positive cells involved in the spread of ovarian cancer

**Fig. 4:**
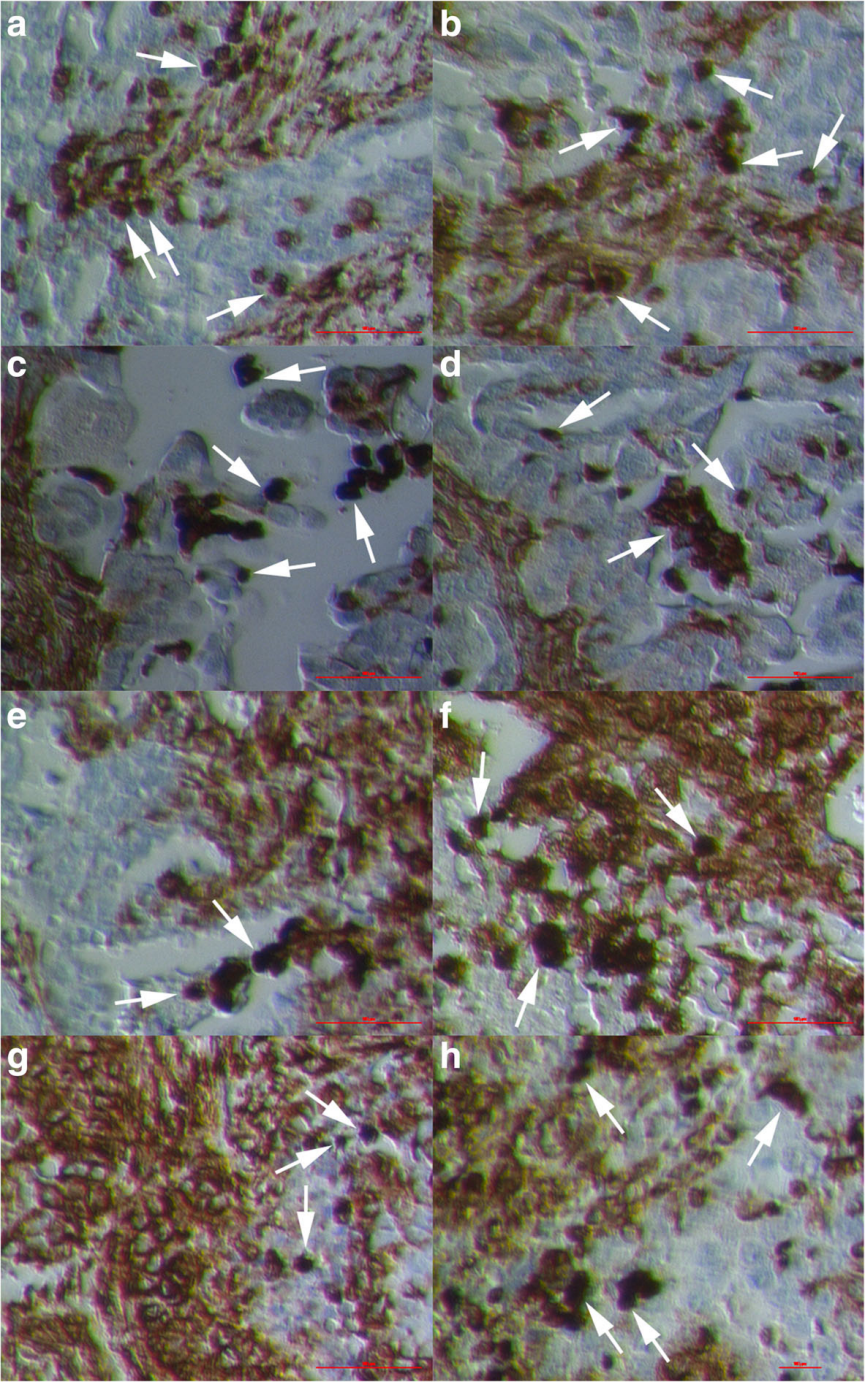
Spreading of cancer tissue by round vimentin-positive cells with diameters of 10–15 μm. In both the region of ovarian sections with early invasion of vimentin-positive cancer tissue (**a**–**d**) and region of later invasion with highly spread cancer tissue (**e**–**h**), the round vimentin-positive cells with diameters of 10–15 μm (arrows) were still present and were changing into the mesenchymal phenotype to possibly spread the cancer tissue. (Inverted microscope, magnifications 100x and 200x). *Legend*: *brown*-vimentin positivity and *blue*-nuclei after HE staining. *Red Bar*: 10 μm for **h** and 100 μm for **a**–**g**

In Fig. 4 and Additional file 2: Figure S2 we can see the spread of vimentin-positive cancer tissue in ovarian sections of women with serous ovarian cancer: earlier spread (Fig. 4a-d and Additional file 2: Figure S2a-d) and later spread (Fig. 4e-h and Additional file 2: Figure S2e-h) of cancer tissue which was positively stained for vimentin. If we look more closely at the pictures, we can see that the spread of cancer tissue was accompanied by clusters of round vimentin-positive cells with diameters of 10–15 μm, which were changing their round shape into the mesenchymal-like phenotype with protrusions and elongation and were possibly involved in the spread of cancer.

### Change of epithelial cells: expression of vimentin

**Fig. 5:**
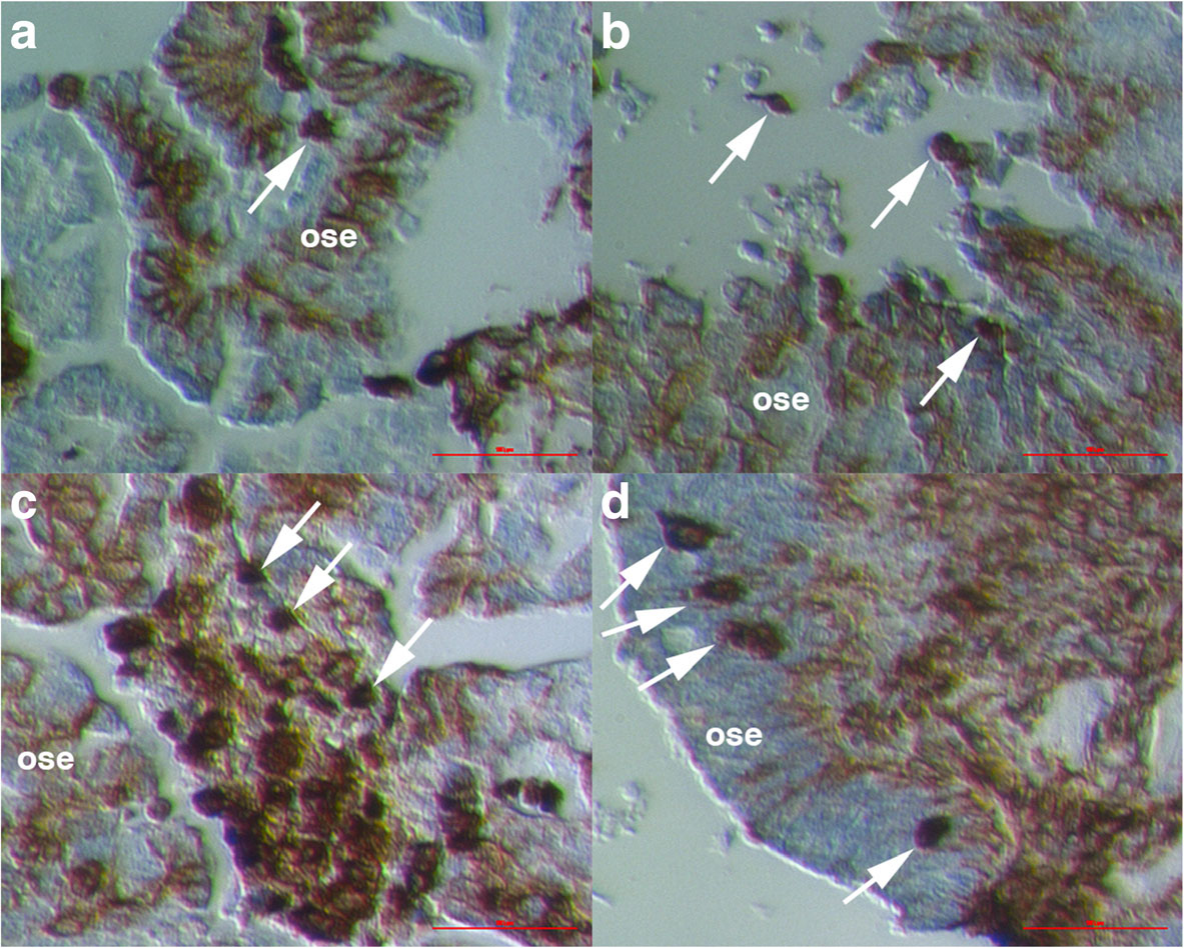
Changed epithelial cells in the OSE layer. At some places the round vimentin-positive cells (arrows) were also present in the OSE layer, which was significantly changed: the epithelial cells expressed cytoplasmic positivity for vimentin (**a**–**c**) or the mesenchymal-like cells formed from them spread among the epithelial cells (**d**). (Inverted microscope, magnification 100x). *Legend*: *brown*-vimentin positivity and *blue*-nuclei after HE staining. *Red Bar*: 100 μm

In some places of the ovarian sections where the round vimentin-positive cells were released from the OSE or invaded the tissue, the nearby epithelial cells in the OSE layer showed a change: the cytoplasmic positivity for vimentin, as can be seen in Fig. 5. The round vimentin positive cells with diameters of 10–15 μm also appeared among the epithelial cells and spread in the tissue by changing their round phenotype into the mesenchymal-like one (Fig. 5d). Otherwise, the epithelial cells in the OSE layer did not express the vimentin positivity.

### Small NANOG-positive cells in the ovarian surface epithelium of women with ovarian serous carcinoma

**Fig. 6:**
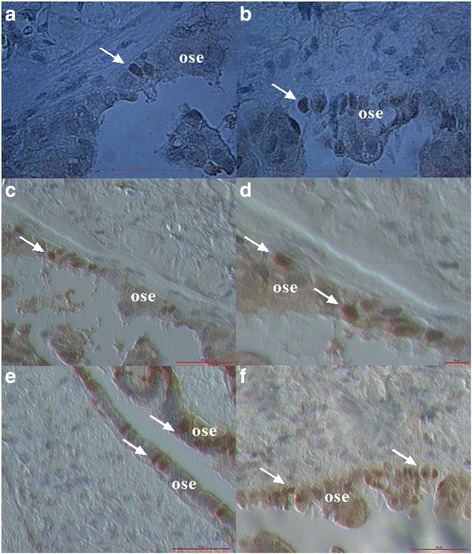
Small NANOG-positive cells (*arrows*) among epithelial cells in the OSE of patients with serous ovarian carcinoma observed by both the light microscope (**a**, **b**) at magnification 400x and inverted microscope (**c**–**f**) at magnifications 100x and 200x. *Legend*: *yellow* to *brown*-NANOG-positivity and *blue*-nuclei after HE staining. *Red Bar*: 10 μm for **d** and 100 μm for **a**–**c**, **f**

Among epithelial cells of the OSE in women with serous ovarian carcinoma, we observed a population of small NANOG-positive cells with diameters of up to 5 μm and nuclei that filled up almost the entire cell volumes, a characteristic of stem cells (Fig. 6). These cells were present among the epithelial cells without any special pattern and were observed by both the light microscope (Fig. 6a and b) and the inverted microscope (Fig. 6c-f). All the cells were positively-stained for marker of pluripotency NANOG because of the nuclei, which spread over whole cell volumes. The NANOG-positivity fits well with the blue staining after HE staining of these cells. In Fig. 6b and f we can see that these small NANOG-positive cells were concentrated in the regions with morphologically changed epithelial cells.

### Bigger round cells with large nuclei positively stained for NANOG

**Fig. 7:**
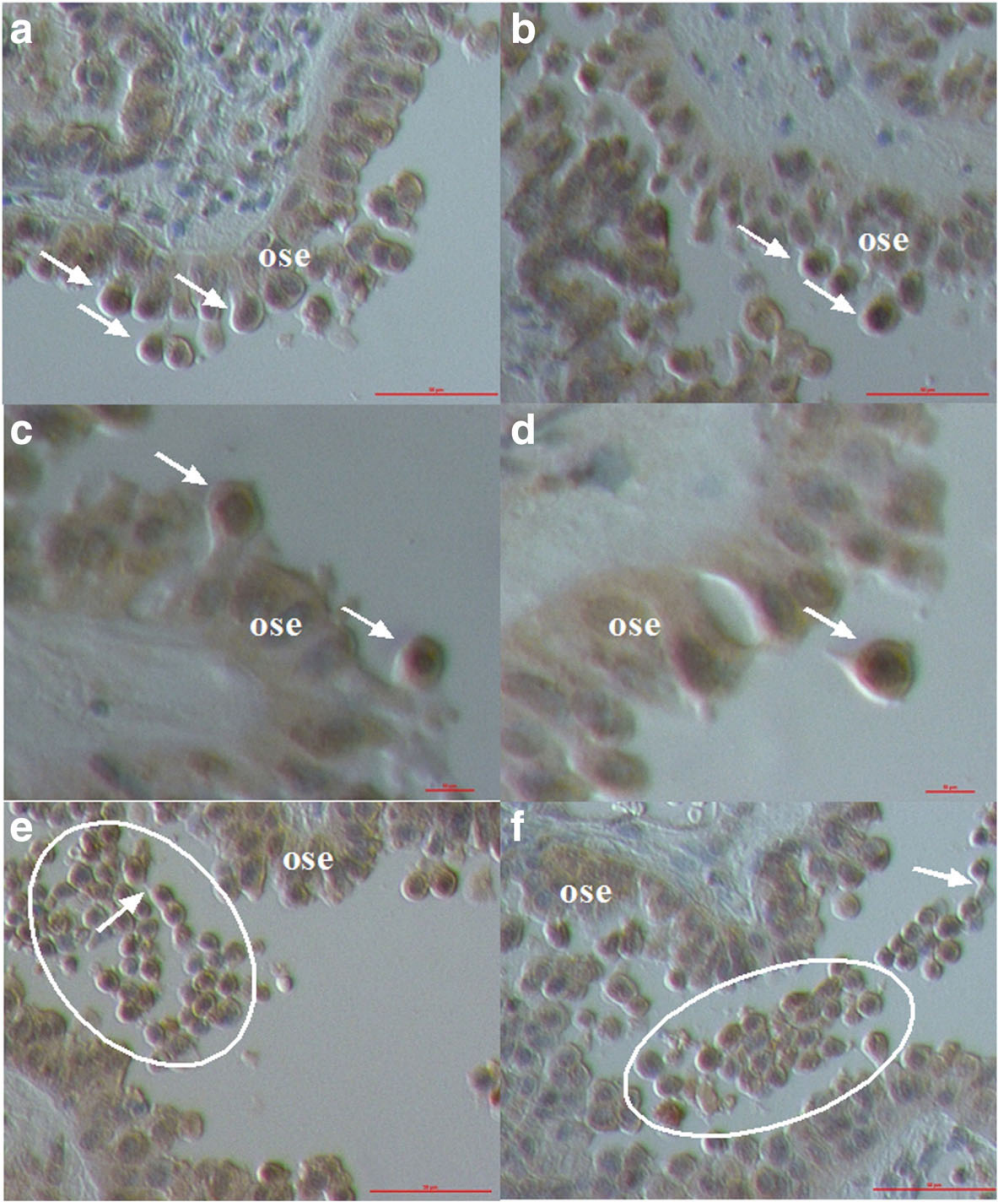
Morphological changes in the ovarian surface epithelium (OSE) of patients with serous ovarian carcinoma: epithelial cells divided and some round cells (*arrows*) were separating from the ovarian surface epithelium (**a**, **b**). Separated round cells had large nuclei filling almost the entire cell volumes (**c**, **d**). *Yellow*-*brown* nuclear staining for NANOG in separated round cells, which nicely conformed to blue HE nuclear staining (**c**, **d**). Separated round cells (circled) were forming cell clusters (**e**, **f**). Some round cells stayed connected by cytoplasmic bridges (arrows) and formed cell clusters (**e**, **f**). (Inverted microscope, magnifications 100x and 200x). *Legend*: *yellow* to *brown*-NANOG positivity and *blue*-nuclei after HE staining. *Red Bar*: 10 μm for **c**, **d** and 100 μm for **a**, **b**, **e**, **f**

The small cells among epithelial cells were not the only population of cells expressing the positive nuclear staining for NANOG. In sections of ovarian tissue of women with ovarian serous carcinoma we observed some typical morphological changes of epithelial cells (Fig. 7). It looked like the epithelial cells were dividing and some round cells sized just above 10–15 μm were separating and releasing from the surface epithelium (Fig. 7a-d). These separated round cells had large nuclei filling almost the whole cell volumes with minimal cytoplasm around them, which is characteristic of stem cells and resembled the round vimentin-positive cells described above. In addition, the separated round cells showed the positive nuclear staining for NANOG, which matched well with the blue HE staining, while the “normal” epithelial cells did not show any positivity for NANOG (Fig. 7a-d). After separation from the surface the epithelium round cells proliferated and formed typical cell clusters (Fig. 7e, f). In these typical cell clusters some of NANOG-positive round cells stayed connected with cytoplasmic bridges (Fig. 7e, f). Interestingly, the presence of such cells was not observed in the ovarian sections of healthy women. We suggest that these cells expressing NANOG may be related to stemness, and it is not excluded that, based on their properties, they are involved in the epithelial-mesenchymal transition in situ.

### A similar population of round cells with large nuclei positively stained for SSEA-4 and SOX2, markers of pluripotency

**Fig. 8:**
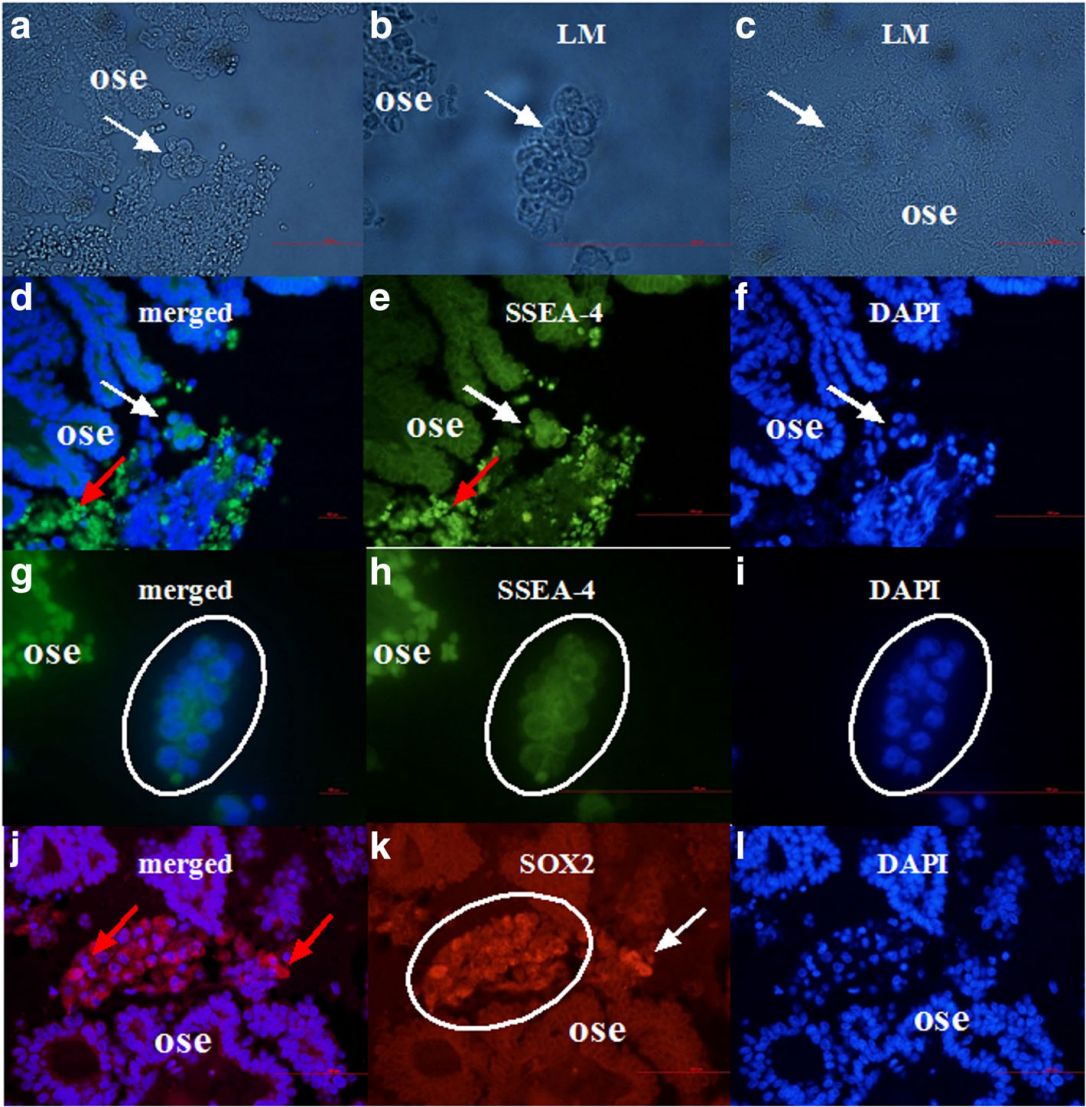
Morphological changes (*white arrows*) in the ovarian surface epithelium (OSE). Round cells with diameters of 10–15 μm were separating from OSE (**a**-**c**). Epithelial cells were dividing and some *green* stained SSEA-4-positive round cells (**e**, *white arrow*) with large blue nuclei after DAPI staining (**f**, *white arrows*) were separating from the ovarian surface epithelium (**d**–**f**, *white arrows*). Separated round cells with large blue nuclei after DAPI staining (**i**) were forming cell clusters (**g**–**i**, circled) expressing SSEA-4 (**h**). Atrophic (autofluorescent) erythrocytes (*red arrows*) were found in the vicinity (**d**, **e**). A similar population of round cells separated from the ovarian surface epithelium (**k**, white arrow), which form cell clusters (**k**, circled) with large blue nuclei after DAPI staining (**l**), expressing SOX2 marker of pluripotency (**k**), was also observed. Atrophic (autofluorescent) erythrocytes (*red arrows*) are found in the vicinity (**j**). (Light microscope: **a**-**c**, magnification 400x; fluorescence microscope: **d**–**l**, magnifications 400x and 1000x). *Legend*: *green*-SSEA4-positivity, *red*-SOX2-positivity, and *blue*-nuclei stained by DAPI. *Red Bar*: 100 μm

Similar morphologic changes of epithelial cells in the ovarian surface epithelium of women with the ovarian serous carcinoma were found after staining ovarian sections for pluripotency-related markers SSEA-4 and SOX2, using immunofluorescence (Fig. 8). After green SSEA-4 staining we could clearly see similar round cells separating from the ovarian surface epithelium, which showed the surface expression of SSEA-4 (Fig. 8d, e); these cells resembled the round vimentin in NANOG-positive cells described above. Round cells had sizes just above 10–15 μm and large nuclei filling almost entire cell volumes with minimal cytoplasm around them, as revealed by DAPI staining (Fig. 8d, f). In addition, separated round cells formed similar cell clusters (Fig. 8g-i). Some comparable findings were found after the red SOX2 staining (Fig. 8j-l); round cells showed nuclear positivity for SOX-2 and formed comparable cell clusters. Atrophic (autofluorescent) erythrocytes were found in vicinity (Fig. 8d, e, j), but they were clearly distinguished from round cells and did not have the nuclei as revealed by DAPI staining (Fig. 8 f, l).

### Co-action of different types of stem cells in the manifestation of ovarian cancer

**Fig. 9:**
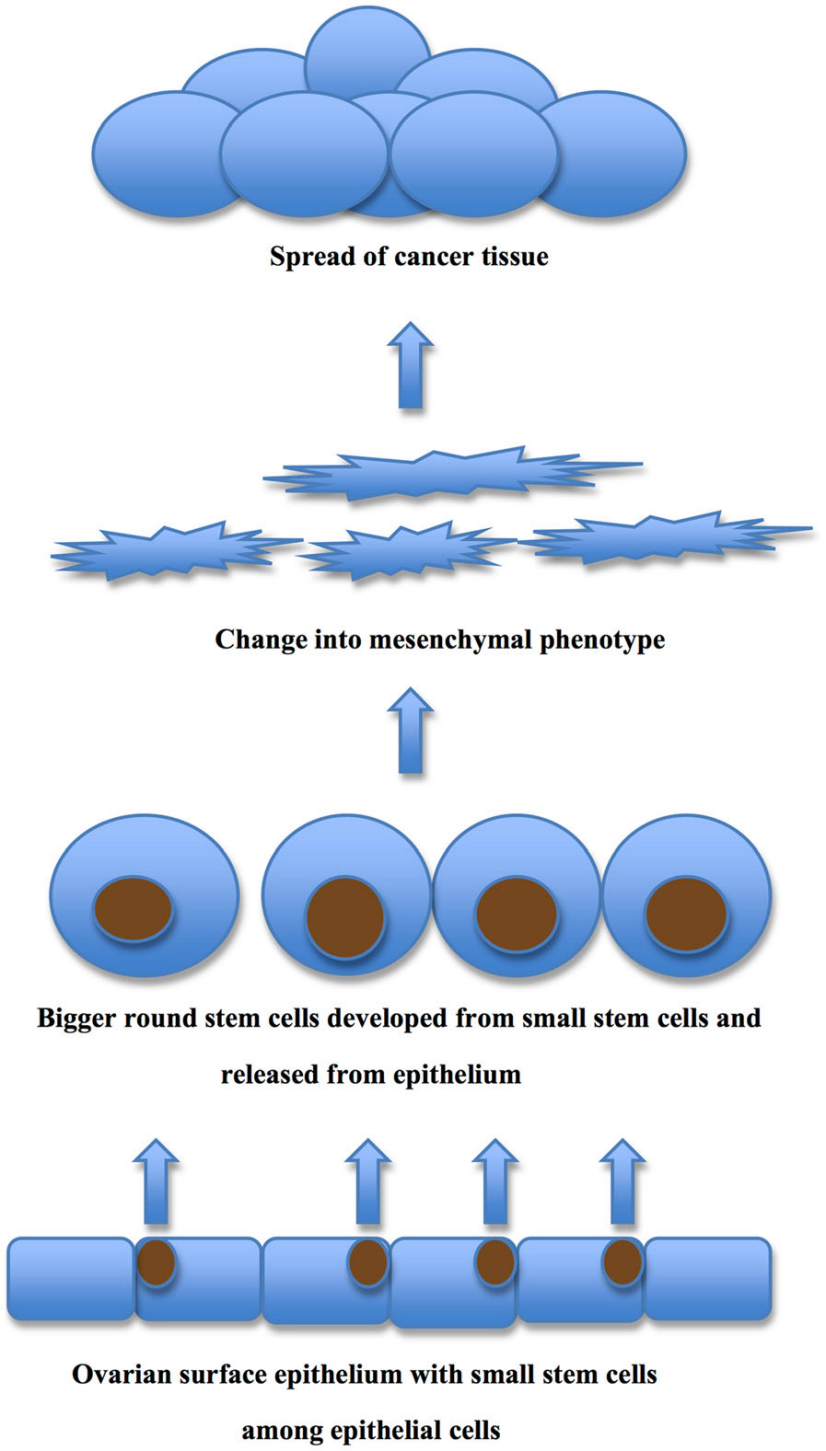
Different populations of vimentin and NANOG-positive (*brown*) putative stem cells in ovarian sections of women with serous ovarian cancer (in situ): small VSEL-like stem cells with diameters of about 5 μm among epithelial cells in the ovarian surface epithelium and bigger round stem cells with diameters of 10–15 μm separating from the layer of epithelial cells and changing into mesenchymal phenotype. Small stem cells might trigger the epithelial-mesenchymal transition or even develop into bigger round stem cells, putative CSCs, and in this way promote the invasion of ovarian cancer

We suggest that the two above mentioned populations of vimentin and NANOG-positive cells: small cells among epithelial cells in the ovarian surface epithelium with diameters of up to 5 μm and bigger round cells with diameters of 10–15 μm separating from epithelial cells are putative stem cells (Fig. 9). It is not excluded that small putative stem cells, which are present among epithelial cells of OSE and concentrate at the morphological changes of epithelial cells or some other factors, initiate the epithelial-mesenchymal transition by their growth and transformation into bigger round cells, positively stained for vimentin and markers of pluripotency NANOG, SOX2, and SSEA-4, which release from the OSE layer, form typical clusters, and invade the ovarian tissues by changing their round phenotype into mesenchymal-like phenotype with protrusions and elongation. We suggest that epithelial-mesenchymal transition doesn’t mean the transition of epithelial cells into mesenchymal cells. More likely, this is a transition of small putative stem cells among epithelial cells into bigger CSCs which are separated from the epithelium and further spread the cancer tissue by their change into the mesenchymal-like phenotype. In spite of that, the epithelial cells are not excluded from this process and support it in an unknown way. Perhaps they somehow embed the small stem cells by their membrane and cytoplasm and then divide or there is an alternative substantiation.

## Discussion

By applying the pluripotency-related marker NANOG, we found two different populations of NANOG-positive cells in sections of ovarian tissue in women with ovarian serous carcinoma: smaller stem cells among epithelial cells in the ovarian surface epithelium and bigger round stem cells releasing from epithelial cells, proliferating and forming typical cell clusters. Very similar populations of cells were positive for vimentin, an important marker of EMT; therefore, we suggest that these two populations of cells are putative stem cells, which might be involved in the epithelial-mesenchymal transition and the manifestation of ovarian cancer.

In general, the origin of CSCs is still poorly understood. There are two generally accepted hypotheses considering the origin of CSCs: the first one is that there are CSCs somatic stem cells that have undergone malignant transformation, and the second one is that more differentiated somatic cells during malignant changes acquire the properties of self-renewal and gain back stems status. In favour of the first hypothesis is the data that normal stem cells, due to active self-renewal and long life span, accumulate genetic mutations that can lead to malignant transformation and development of CSCs. On the other hand, it was shown on experimental cancer models that CSCs are not necessarily derived from normal stem cells [31].

In our study, we identified small putative stem cells among epithelial cells of the ovarian surface epithelium with diameters of up to 5 μm, which expressed the pluripotency-related marker NANOG. These small putative stem cells resembled very small embryonic-like stem cells (VSELs) from some other human adult tissues and organs such as bone marrow [32] and umbilical cord blood [33]. The VSELs are proposed to persist as dormant in adult human tissues and organs from the embryonic period of life to regenerate the damaged tissues and may be involved in the manifestation of cancer at an inappropriate condition in the body [34]. Very similar cells have already been found in the ovarian surface epithelium of healthy adult human ovaries [35–38]. In this study, the immunocytochemistry showed that these small VSEL-like stem cells expressed cytoplasmic positivity for vimentin and possibly grew into bigger round cells which were strongly positive for vimentin. In this way, perhaps the small VSEL-like stem cells activated themselves and developed into CSCs.

The observation of tumor sections also showed morphological changes of epithelial cells in terms of malignant transformation. We noticed that specific round cells were being released from OSE in patients with ovarian serous carcinoma. The separated round cells were about 10–15 μm in size and had large nuclei, filling almost the whole cell volume, which is the characteristic of stem cells. The separating cells were NANOG-positive, while the OSE was not. Moreover, the similar population of round cells was also stained positively for EMT-related marker vimentin and other markers of pluripotency, such as surface antigen SSEA-4 and nuclear SOX2, thus further indicating that these cells may be stem cells involved in EMT. After the separation of NANOG-positive round cells from the surface epithelium those cells proliferated and formed clusters of identical cells, which is another characteristic of stem cells. In cell clusters, some of these round NANOG-positive cells were still connected by cytoplasmic bridges. Interestingly, cytoplasmic bridges may be found also among female germ cells – oogonia – which are similarly characterized by the round shape and nuclei filling the whole cell volumes [39]. EMT induced in pathologic conditions has already been associated with the generation of CSC-like cells [21]. We suggest that bigger NANOG-and vimentin-positive round cells with large nuclei that separated from surface epithelium represent putative cancer stem cells (CSCs) which arise in the EMT process. These cells proliferated and formed typical cell clusters of identical cells, which might represent the beginning of tumor formation and invasion. First we thought that these cells were originating from epithelial cells (e.g., by their transdifferentiation), but our data show that these cells possibly originate from small VSEL-like stem cells, which are present among epithelial cells in the OSE.

The positivity of big round cells – putative cancer stem cells – for both NANOG and vimentin markers is not a surprise as it has already been found that the transcription factor NANOG regulates the epithelial-mesenchymal transition and chemoresistance through activation of the STAT3 pathway in epithelial ovarian cancer [16]. The EMT, a basic process in the morphogenesis of fetal tissues during embryogenesis and in normal adult tissues during regeneration, is defined as loss of epithelial traits of former epithelial cells and acquisition of mesenchymal characteristics [20]. EMT in epithelial tumor cells increases the cell invasion capacity and enhances their degree of malignancy [22, 23]. The characteristic of EMT: increasing cell motility and invasion [40, 41], metastases and resistance to drugs [42, 43], are similar to characteristic of the high grade serous carcinoma: invasion, dissemination, metastases, resistance to chemotherapy and recurrence of disease. Based on our data, we suggest that big round cells expressing vimentin and a degree of pluripotency, released from OSE and formed typical cell clusters, invaded the tissues and spread the malignancy by change of their round shape into mesenchymal-like phenotype with protrusions and elongation.

The EMT process in malignant cells can be triggered by different signals, some of them expressed by tumor stroma [44] including transforming growth factor-ß [45, 46]. Epidermal growth factor receptor signaling caused ovarian cancer cells to resist cisplatin and to recur because of the EMT process [47]. The cisplatin-induced EMT has been found to increase the expression of CSC markers (NANOG and others) and mesenchymal markers (N-cadherin, vimentin) and decrease the expression of epithelial markers (E-cadherin) [48]. Our data show that it is not excluded that small putative stem cells expressing NANOG, which were present among epithelial cells, somehow in some condition trigger the EMT process by growing into bigger vimentin and NANOG-positive cells through which CSCs are generated. We observed that these small NANOG-positive cells were more concentrated at places of morphologically changed epithelial cells and release of typical round cells.

Our data indicate that the process of EMT may be identified in situ on a tumor tissue and this observation, based on application of vimentin and stemness/pluripotency related markers. Maybe this phenomenon could serve as an important in situ prognostic marker of ovarian cancer in the future. The knowledge about CSCs and their critical role not only in cancer development but also in resistance to drugs and recurrence of disease opens a new possible way to tumor treatment. CSCs and EMT as the mechanism of development of CSCs can present a new target for novel drugs for epithelial ovarian cancer treatment. Although many questions regarding CSCs and EMT still remain, further research in the field of isolation and in vitro cultivation of cell cultures as well as in situ observation of tumor slides may give some additional answers.

## Conclusions

The process of EMT has until now been mostly studied in ovarian cancer cell cultures in vitro. Our findings show the EMT as a process also appearing in vivo because we identified it in situ: in tumor sections of the high grade serous ovarian carcinoma. For the first time, we show a population of putative CSCs, which were present in situ: round cells with diameters of 10–15 μm which were positively stained for vimentin, NANOG, and other markers of pluripotency, were released from the ovarian surface epithelium, formed typical cell clusters, and were able to invade the tissue by change of their round shape into mesenchymal-like phenotype with protrusions and elongation. These cells very possibly originate from small VSEL-like stem cells, which were present among epithelial cells in ovarian surface epithelium and have already been related to ovarian cancer [49]. Our results better elucidate the CSCs and indicate that further research of CSCs and EMT in situ is needed in order to better understand the mechanisms of the ovarian cancer. Extended knowledge about CSCs and EMT would help in a better diagnosis of ovarian cancer. CSCs and EMT might also be seen as a possible target for novel drugs in treatment of epithelial ovarian cancer that would improve the prognosis of this deadly disease.

## Additional files

Additional file 1: Figure S1. Round vimentin-positive cells (*arrows*) with diameters of 10–15 μm being released from the ovarian surface epithelium (OSE). (Light microscope, magnification 400x). *Legend*: *brown*-vimentin positivity, *blue*-HE stained nuclei. *Red bar*: 100 μm. (JPG 1069 kb)
Additional file 2: Figure S2. Spreading of cancer tissue by round vimentin-positive cells with diameters of 10–15 μm. In both the region of ovarian sections with early invasion of vimentin-positive cancer tissue (a-d) and region of later invasion with highly spread cancer tissue (e-h), the round vimentin-positive cells with diameters of 10–15 μm (*arrows*) were still present and were changing into the mesenchymal phenotype to possibly spread the cancer tissue. (Inverted microscope, magnifications 100x and 200x). *Legend*: *brown*-vimentin positivity and *blue*-nuclei after HE staining. *Red Bar*: 10 μm for f and 100 μm for a-e, g, h. (JPG 2004 kb)

## Abbreviations

CSC: Cancer stem cell
DAPI: 4',6-diamidino-2-phenylindole
EMT: Epithelial-to-mesenchymal transtion
ESC: Embryonic stem cell
FITC: Fluorescein isothiocynate
HE: Hematoxylin and eosin
IHC: Immunohistochemistry
NANOG: Nanog homeobox
OCT4: Octamer-binding protein 4
OSE: Ovarian surface epithelium
PBS: Phosphate buffered saline
PE: Phycoerythrin
SOX2: SRY-box 2
SSEA-4: Stage-specific embryonic antigen 4
STAT3: Chemoresistance through activation of the signal transducer and activator of transcription 3
TMA: Tissue microarray
VSELs: Very small embryonic-like stem cells
