# Supplementary figures and images for: Putative stem cells and epithelial-mesenchymal transition revealed in sections of ovarian tumor in patients with serous ovarian carcinoma using immunohistochemistry for vimentin and pluripotency-related markers
Source: J Ovarian Res. 2017 Feb 23;10:11. doi: 10.1186/s13048-017-0306-7 (PMC5324304; doi:10.1186/s13048-017-0306-7)

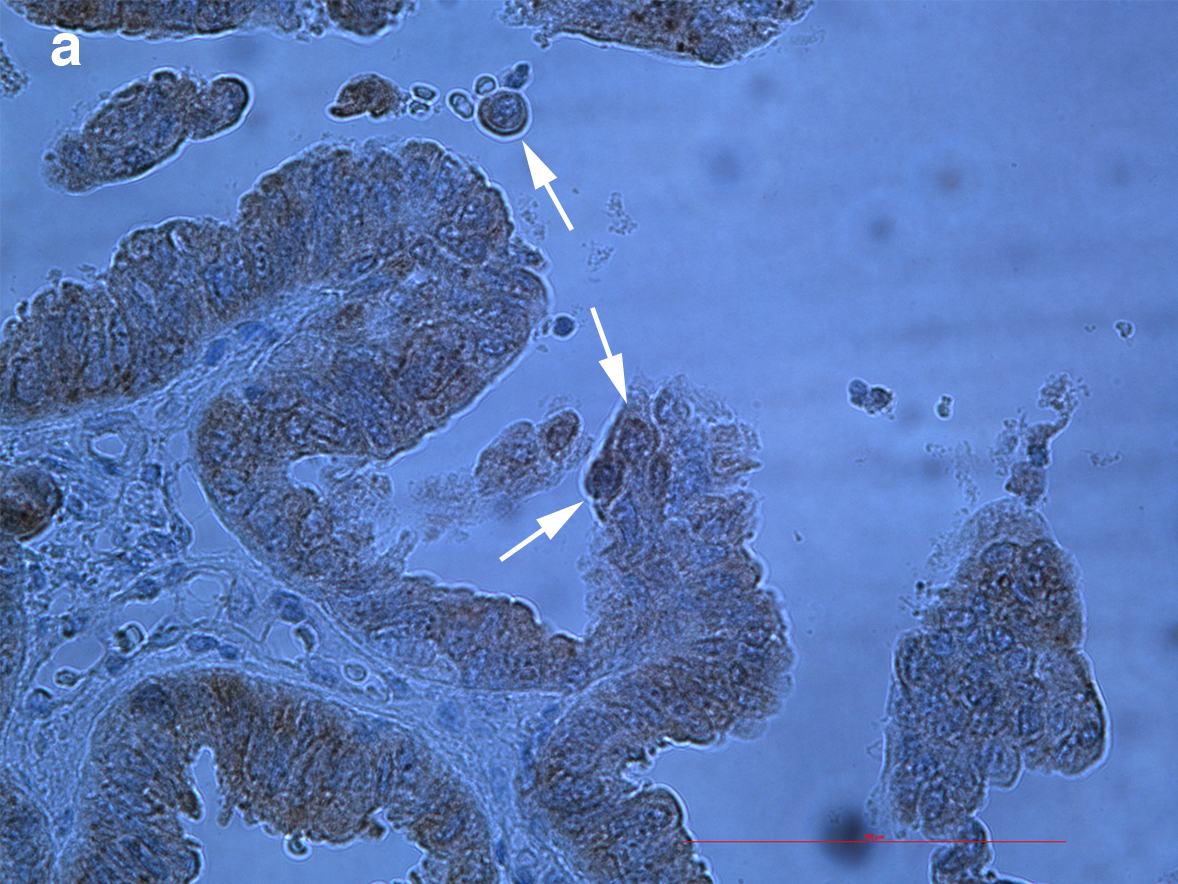

Supplement: Additional file 1: Figure S1. — Round vimentin-positive cells (arrows) with diameters of 10–15 μm being released from the ovarian surface epithelium (OSE). (Light microscope, magnification 400x). Legend: brown-vimentin positivity, blue-HE stained nuclei. Red bar: 100 μm. (JPG 1069 kb) [file 13048_2017_306_MOESM1_ESM.jpg]

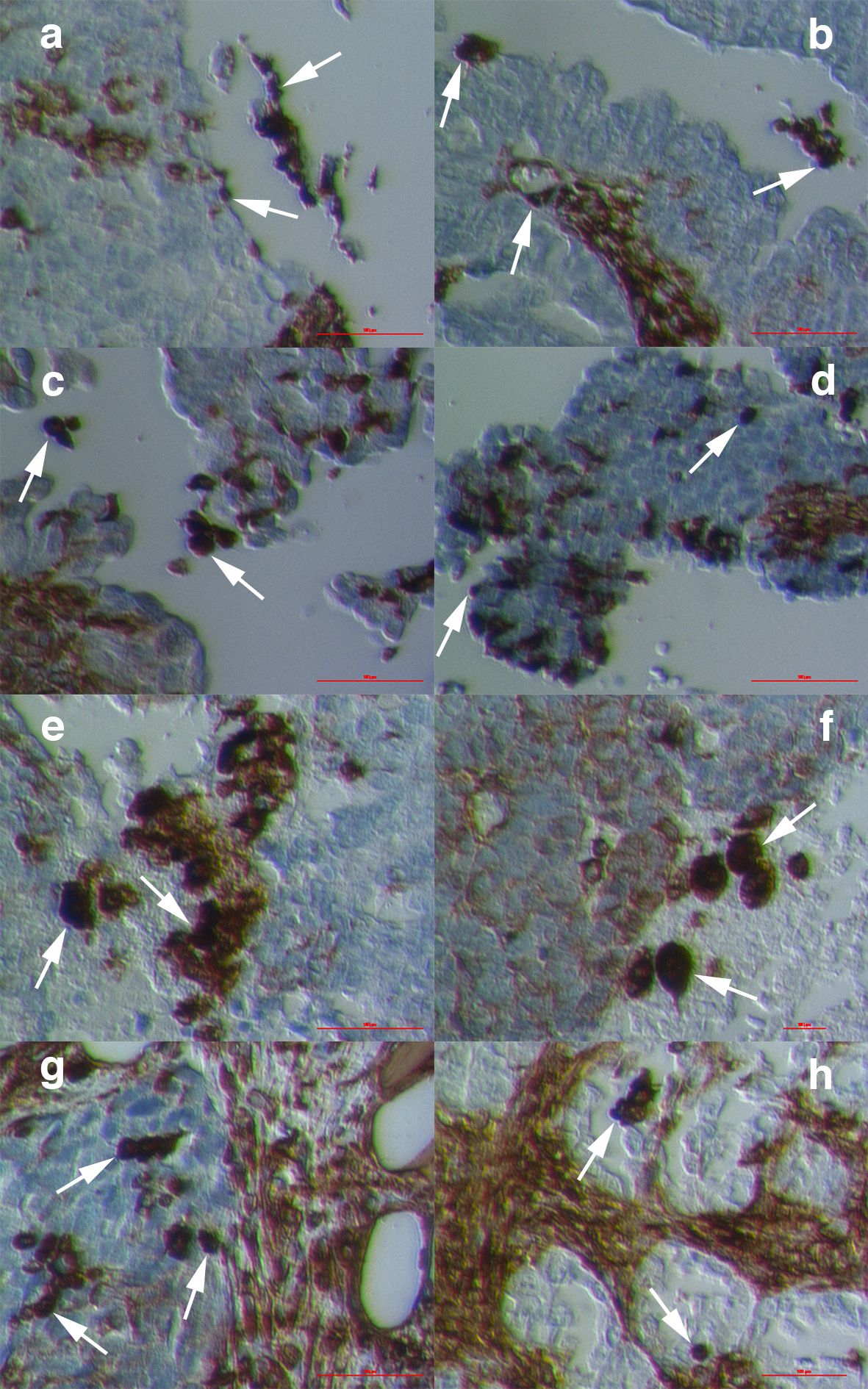

Supplement: Additional file 2: Figure S2. — Spreading of cancer tissue by round vimentin-positive cells with diameters of 10–15 μm. In both the region of ovarian sections with early invasion of vimentin-positive cancer tissue (a-d) and region of later invasion with highly spread cancer tissue (e-h), the round vimentin-positive cells with diameters of 10–15 μm (arrows) were still present and were changing into the mesenchymal phenotype to possibly spread the cancer tissue. (Inverted microscope, magnifications 100x and 200x). Legend: brown-vimentin positivity and blue-nuclei after HE staining. Red Bar: 10 μm for f and 100 μm for a-e, g, h. (JPG 2004 kb) [file 13048_2017_306_MOESM2_ESM.jpg]
